# Affinity Capture Enrichment versus Affinity Depletion: A Comparison of Strategies for Increasing Coverage of Low-Abundant Human Plasma Proteins

**DOI:** 10.3390/ijms21165903

**Published:** 2020-08-17

**Authors:** Nicolai Bjødstrup Palstrøm, Lars Melholt Rasmussen, Hans Christian Beck

**Affiliations:** 1 Centre of Individualized Medicine in Arterial Diseases (CIMA), Odense University Hospital, DK-5000 Odense C, Denmark; Nicolai.Bjodstrup.Palstrom@rsyd.dk (N.B.P.); lars.melholt.rasmussen@rsyd.dk (L.M.R.); 2 Centre for Clinical Proteomics, Department of Clinical Biochemistry and Pharmacology, Odense University Hospital, DK-5000 Odense C, Denmark

**Keywords:** affinity-based enrichment, plasma proteomics, immunodepletion, protein equalization

## Abstract

In the present study, we evaluated four small molecule affinity-based probes based on agarose-immobilized benzamidine (ABA), O-Phospho-L-Tyrosine (pTYR), 8-Amino-hexyl-cAMP (cAMP), or 8-Amino-hexyl-ATP (ATP) for their ability to remove high-abundant proteins such as serum albumin from plasma samples thereby enabling the detection of medium-to-low abundant proteins in plasma samples by mass spectrometry-based proteomics. We compared their performance with the most commonly used immunodepletion method, the Multi Affinity Removal System Human 14 (MARS14) targeting the top 14 most abundant plasma proteins and also the ProteoMiner protein equalization method by label-free quantitative liquid chromatography tandem mass spectrometry (LC-MSMS) analysis. The affinity-based probes demonstrated a high reproducibility for low-abundant plasma proteins, down to picomol per mL levels, compared to the Multi Affinity Removal System (MARS) 14 and the Proteominer methods, and also demonstrated superior removal of the majority of the high-abundant plasma proteins. The ABA-based affinity probe and the Proteominer protein equalization method performed better compared to all other methods in terms of the number of analyzed proteins. All the tested methods were highly reproducible for both high-abundant plasma proteins and low-abundant proteins as measured by correlation analyses of six replicate experiments. In conclusion, our results demonstrated that small-molecule based affinity-based probes are excellent alternatives to the commonly used immune-depletion methods for proteomic biomarker discovery studies in plasma. Data are available via ProteomeXchange with identifier PXD020727.

## 1. Introduction

Human body fluids like serum, plasma, or cerebrospinal fluids are accessible in a clinical context and carry a large number of proteins that are regarded as highly informative about physiological and pathological states. Therefore, these body fluids are thought to contain novel protein biomarkers that may become useful for disease diagnosis and prognosis, or in precision medicine for individualized treatment or in drug development.

Although very different in total protein concentrations—spanning from 70 mg/mL for plasma to less than 1/100 of this concentration for cerebrospinal fluid [1,2]—the protein composition of these fluids is comparable and therefore they share similar challenges in relation to protein biomarker discovery, namely the sample complexity and especially a dynamic concentration range of proteins that spans over more than 10 orders of magnitude. The low-abundant proteins (LAPs)—present in the ng-to-pg per mL range in plasma—are regarded to be the most attractive ones as promising biomarker candidates, mainly because they may reflect a leakage of proteins from relevant tissues, e.g., a tumor, into the blood stream. Their discovery by analytical techniques is, however, hampered by the presence of the very high-abundant classical plasma proteins like serum albumin, immunoglobulins and proteins of the complement system. These proteins make up more than 95% of the protein content of human plasma [2]. Therefore, a great effort is made to reduce sample complexity in biomarker discovery research employing a variety of methods. These include depletion of high-abundant proteins using immune affinity approaches, selective capturing of sub-proteomes [3,4,5], protein corona formation on nanoparticles [6] or enrichment techniques alone [7,8] and/or in combinations with extensive fractionation techniques such as sodium dodecyl sulphate polyacrylamide gel electrophoresis (SDS-PAGE) [9], strong cation exchange (SCX) [10], isoelectric focusing (IEF) [11], hydrophilic interaction chromatrography (HILIC), and high-pH separation [9]. Despite these efforts to reduce sample complexity, plasma biomarker studies generally identify a significantly lower number of proteins compared to proteomic analysis of protein extracts from tissue biopsies or isolated from cell cultures, despite the fact that more than 10,000 proteins to date have been identified in plasma, according to the Plasma Proteome Database (http://www.plasmaproteomedatabase.org, last access day: 2 February 2015) [12,13]. One likely explanation for this is, however, that depletion and fractionation do not eliminate the “order of magnitude”—a challenge, that one is inevitably linked to when analyzing the plasma proteome—but instead they just shift this obstacle down the concentration ladder of this proteome. Moreover, many of these techniques for sample complexity reduction are laborious and expensive, thereby hampering their application in a clinical context that most often requires the analysis of hundreds of samples.

Therefore, there is still a great need for new experimental strategies that targets the LAP plasma proteome using simple, fast, and inexpensive sample preparation procedures. Thus, in the present study, we compared four different affinity enrichment strategies for the selective capturing of plasma sub-proteomes, a protein equalization technique and a newly developed immune-depletion system that depletes high-abundant proteins (HAPs) and moderately abundant proteins (MAPs) with one of the most frequent used top 14 HAP immune depletion systems.

## 2. Results

We head-to-head compared used methods for the depletion of abundant proteins in plasma samples, the MARS 14 cartridge and the Proteominer immobilized peptide library with four affinity-based approaches, based on approaches based on cAMP-, ATP-, pTYR, or ABA linked to agarose beads. Specifically, the impact of each of the tested methods on the number of proteins identified in six replicate analysis of a plasma sample by data dependent nano-LC-MSMS analysis and their depletion/enrichment capabilities as judged by label free quantification was tested. Moreover, the reproducibility of each method for both high-abundant and low-abundant plasma proteins was assessed by correlation analyses of the six replicate experiments (see Supplementary Figure S1 for overview).

### 2.1. Head-to-Head Comparison of Plasma Proteome Coverage

**Figure 1:**
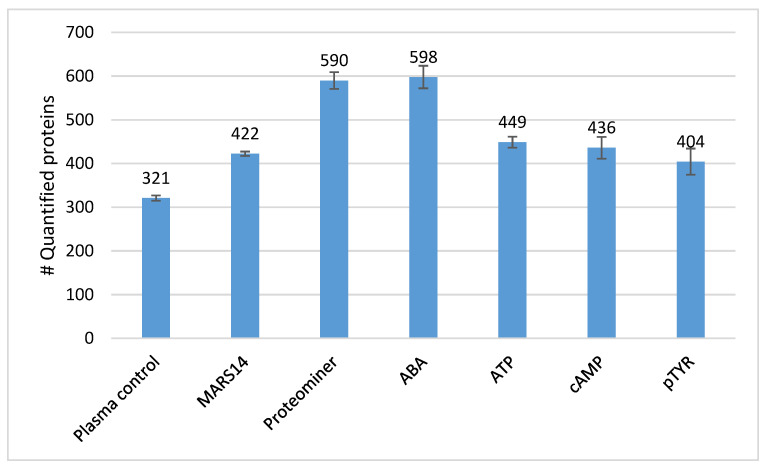
Number of proteins identified by the immunodepletion methods, the protein equalization method, and the affinity-based purification methods using nano-liquid high performance chromatography combined with tandem mass spectrometry (LC-MSMS) analysis. Data are mean values of six replicate experiments and the error bars indicate standard deviation.

Nano-LC-MSMS analyses of the six replicate un-depleted plasma samples identified an average of 321 proteins, whereas the corresponding analysis of the plasma samples depleted for 14 most abundant plasma proteins using the MARS14 depletion column or the Proteominer protein equalization method, identified an average of 422 proteins or 590 proteins (Figure 1), which is an increase of 18% or 70% in protein identifications compared to the analysis of un-depleted plasma. Among the affinity-based approaches, the lowest number of unique proteins identified was done with the pTYR-based affinity approach (404 unique proteins), whereas the highest number of proteins were identified with the ABA-based affinity approach that identified an average of 598 unique proteins (Figure 1), an improvement of 55% in the number of identified proteins as compared to the analysis of the un-depleted plasma sample. The ATP-based affinity approach and the cAMP-based affinity approach performed equally well and identified an average of 449 and 436 unique proteins, respectively.

### 2.2. Comparison of the Efficiency of the Removal of High Abundant Plasma Proteins

Next, we compared the capability of immuno-depletion and protein equalization with the affinity-based approaches ABA, cAMP, pTYR, and ATP to remove the most abundant proteins from plasma as determined by label free quantitative analysis, as described in the methods section (Table 1). In general, The Proteominer equalization method performed generally slightly less efficiently than the MARS14 cartridge, especially for apolipoprotein A1, IgMH and fibrinogen alpha chain whereas all four of the affinity-based probes, with the exception of IgMH, demonstrated excellent removal of high-abundant proteins from plasma.

### 2.3. Comparison of the Reproducibility of Affinity-Based Proteomic Enrichment

**Figure 2:**
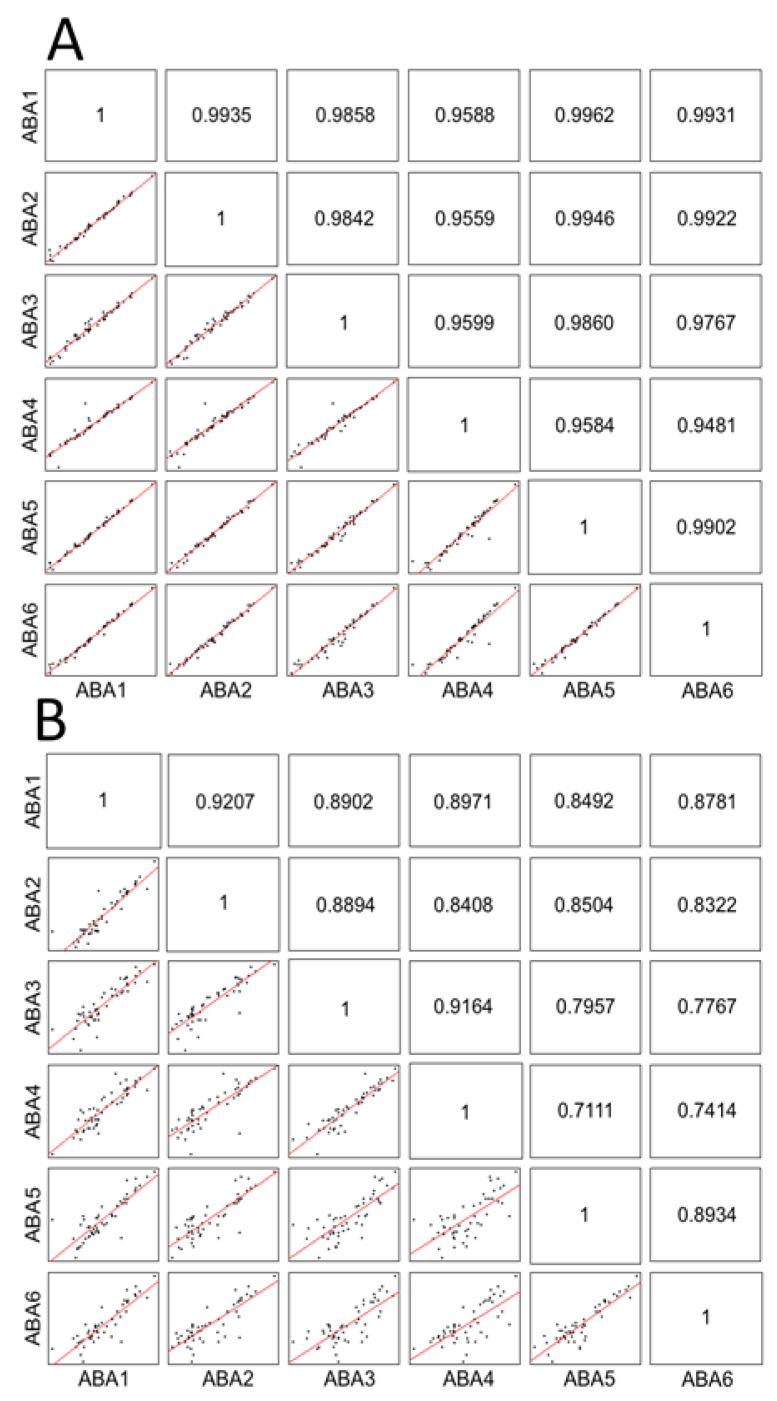
Linear correlation analysis of six replicates for the ABA affinity-based experiment for the (**A**) 50 most abundant proteins and the (**B**) 50 least abundant proteins quantified by nano-LC-MSMS analysis and precursor ion area detection. The concentrations of the measured proteins in plasma were based on values extracted from the plasma proteome database (http://www.plasmaproteomedatabase.org). Depicted data are log2-transformed.

We then evaluated the reproducibility of the tested methods by performing linear correlation analyses of pairs of all combinations of the six replicate experiments yielding a total of 15 correlations for each of the tested methods (Figure 2 and Table 2).

As illustrated in Figure 2, the ABA-based affinity method demonstrates excellent correlations for all combinations of the six replicate experiments for the 50 most abundant proteins detected (Figure 2A) and also fairly good correlations for the 50 lowest abundant proteins detected with this method. Table 2 summarizes the means of the linear correlation analysis of the 50 most abundant plasma and 50 least abundant plasma proteins for all tested methods. All methods performed equally well for the analysis of the most abundant plasma proteins and showed correlation coefficients of 0.93 or higher. For the analysis of low-abundant plasma proteins, superior linear correlations were observed for the cAMP-affinity based method and the MARS14 immunodepletion methods whereas the Proteominer protein equalization method and the ATP-, pTYR-, ABA-affinity-based methods demonstrated reasonable linear correlations ranging from 0.83 to 0.85.

### 2.4. Qualitative Comparison

**Figure 3:**
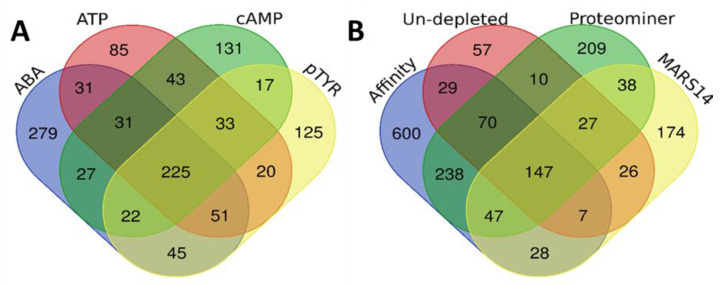
Unique and shared proteins identified in (**A**) samples enriched for low-abundant proteins using the affinity-based probes pTYR, ABA, ATP, or cAMP, and in (**B**) un-depleted plasma samples, plasma samples depleted for high abundant proteins using the MARS14 depletion column, the Proteominer protein equalization method, and the sum of proteins identified as identified in (**A**).

Qualitative comparison of the affinity-based enrichment and the immune depletion methods is shown in Figure 3. A total of 1165 unique proteins were identified with the affinity-based methods (Figure 2A) whereas the ABA-based method demonstrated a large degree of complementarity by the identification of 279 proteins that were only detected with this method. Moreover, the ATP-, pTYR and cAMP-based methods showed some degree of complementarity by the identification of 85, 125, and 131 proteins unique for each of these methods. When comparing the unique proteins identified with the affinity-based methods with the unique proteins identified with the MARS14 and the Proteominer methods, a pronounced complementarity is observed with the affinity-based methods as 600 out of 1707 unique proteins identified across all methods.

## 3. Discussion

Human plasma consists of approximately 20 proteins that account for more than 99% of the protein content [2] and several attempts to circumvent the influence of these high-abundant proteins on the analysis of medium-to low-abundant proteins have been made. The most often used methods include immunodepletion methods that target the top 14 abundant proteins such as the Multi-Affinity Removal System (MARS14) Human 14 column or the Seppro IgY-14 column [14], but also the Seppro IgY Supermix column that is designed to deplete the ~50 moderately abundant proteins in addition to the top 12–14 most abundant proteins in plasma [15]. Another frequently used method is the ProteoMiner method which is based on the limited and equivalent binding capacity of a hexapeptide library for every protein leading to an enrichment of low-abundant proteins by washing away the high-abundant proteins. By contrast, affinity capture enrichment of low-abundant plasma proteins using small-molecule based affinity baits have almost not been applied for the proteomic profiling of low-abundant plasma proteins despite the fact that versions of this principle have become the method-of-choice for the proteomic profiling of low-abundant protein kinases in drug target studies [16,17]. We envisaged that the principle of affinity capturing of proteins of lower abundances by using immobilized small molecule baits that target sub-proteomes of the plasma proteome is a viable alternative to the existing methods for increasing coverage of plasma proteome analysis and detecting lower abundant proteins in plasma. Therefore, in the present study we aimed to test four affinity-based protein purification approaches for the removal of high abundant proteins and the capture and enrichment of lower abundant proteins from plasma, and to make a head-to-head comparisons with the commonly used methods for removing high-abundant plasma proteins, prior to proteome analysis, the MARS14 immune-depletion and the ProteoMiner protein equalization method. The affinity-based methods tested for protein enrichment were based on agarose-immobilized ATP, cAMP, p-TYR, or ABA. All affinity probes tested are commercially available and were selected based on their complementary affinity towards different proteins and protein groups. The ATP-based affinity probe has been applied for the isolation of ATP-binding proteins such as protein kinases [18] and a variety of other protein groups [19], whereas the cAMP-based affinity probe has a high affinity towards proteins that contain a cyclic nucleotide binding domain (CNBD) [20]. These proteins include cAMP-dependent protein kinase A proteins [21,22], cyclic nucleotide gated ion channels [23], and guanine nucleotide exchange factors [24]. By contrast, the pTYR based affinity probe is supposed to enrich for phosphor-amino acid binding proteins such as phosphatases and SH-domain-containing proteins [25] The ABA-based probe enriches for trypsin-like proteases including thrombin, kallikrein, the former being a key enzyme in the blood coagulation cascade [4].

In general, we found that all affinity-based methods removed the majority of the 14 most abundant proteins from plasma more efficiently than the MARS14 and the ProteoMiner methods (Table 1), except for the removal of IgMH. Especially the removal of albumin—by far the most abundant plasma protein—by the affinity capture methods was highly efficient and comparable with the results obtained with the MARS14 and the ProteoMiner methods. This efficient removal of high-abundant proteins by the affinity-based methods leads to the expectation that a higher number of proteins will be identified by LC-MSMS in these samples, as compared to the reference sample, due to a lower sample complexity. In fact, we found the ABA-, ATP-, and the cAMP-based affinity capture methods provided significantly more protein identifications as compared to the reference sample (Figure 1). The MARS14 and ProteoMiner methods also provided a markedly higher number of protein identifications as compared to the reference samples, in line with previous observations [26]. Interestingly, although a comparable number of proteins were identified by all the methods, the complementarity of the affinity capture methods was striking close to 35% for all of the identified proteins that were detected with these methods. This may be explained by fact that affinity-based methods selectively enrich the plasma sample for less abundant proteins that have a higher affinity for the bait in question, whereas these proteins—in the absence of a high affinity capture—will tend to bind to very high-abundant plasma proteins, such as immunoglobulins and albumin, even if the affinity of the less abundant proteins for high abundant ones is low [27]. Consequently, the less abundant proteins will be removed or sequestered to even lower concentrations when removing high-abundant proteins using immune depletion or protein equalization and are therefore not detectable by MS with these methods. In fact, when comparing protein signal intensities throughout the plasma concentration range (Supplementary Table S2) it becomes clear that relatively few proteins are detected with similar signal intensities across the six methods tested, whereas predominantly less abundant proteins were detected with the affinity capture methods but not the MARS14 method.

The overall goal with most clinical proteomic discovery studies is the measurement of potential disease-specific or treatment-specific concentration changes of—per se—low-abundant proteins in a reasonable number of patient samples. Important analytical parameters that should be considered in clinical proteomics include the reproducibility (analytical variation) of the data, and to some extent, the sensitivity of the analytical method applied. As demonstrated by the correlation analysis of the data from individual label-free quantitative LC-MSMS analysis of the six replicate samples prepared by the affinity-based methods a very high degree of reproducibility for proteins present at both high and low concentrations in plasma was observed with these methods–also in line with the results obtained by the MARS14 depletion method and the Proteominer protein equalization method. This indicates that the technical variation (sum of the pre-analytical variation and the analytical variation) for the tested methods is rather low, even for plasma proteins present in low concentrations thereby underlining the usefulness of the tested methods in plasma biomarker studies, as already demonstrated in our recent study [28].

## 4. Material and Methods

### 4.1. Plasma Samples and Reagents

Human plasma samples were obtained from 100 healthy blood donors at Odense University Hospital, Odense, Denmark. Trypsin was kindly provided by NovoNordisk A/S (Gentofte, Denmark). Agarose-immobilized O-Phospho-L-Tyrosine, 8-Amino-hexyl-cAMP, and 8-Amino-hexyl-ATP were obtained from Jena Bioscience GmbH (Jena, Germany). The Proteominer Protein Enrichment Kit was obtained from Biorad Corporation, Hercules, CA, USA. Multi Affinity Removal System (MARS) Human 14 depletion spin column was obtained from Agilent Technologies (Palo Alto, CA, USA), and depletes plasma samples of 14 high-abundant proteins.

### 4.2. High-Abundant Protein Immunodepletion, Affinity-Based Enrichment and Protein Equalization

The depletion procedures using the MARS14 spin column was performed according the manufactures description. Briefly, for MARS14 depletion 7 µL plasma was diluted and applied to the pre-equilibrated spin column. The collected flow-troughs were concentrated with a 3 kDa spin filter and the concentrated protein samples were re-dissolved in 0.2 M tetraethyl ammonium bicarbonate (TEAB) and collected for further processing as described below. Protein equalization using the ProteoMiner Enrichment Kit was performed according to the manufacturer’s description with minor modifications. Briefly, plasma samples were applied to beads with different hexapeptide ligands with affinity for specific proteins. After incubation, proteins in excess are removed by washing and bound proteins are eluted by sequential incubation in 8 M urea, desalted and transferred to a 0.2 M TEAB solution as described for the immunodepletion experiment. Affinity-based enrichment, using the agarose-immobilized benzamidine (ABA), O-Phospho-L-Tyrosine (pTYR), 8-Amino-hexyl-cAMP (cAMP), and 8-Amino-hexyl-ATP (ATP) agarose probes, was performed by incubating 20 µL of the probe slurry with 1 mL of a 100 µL plasma sample diluted with 900 µL PBS with 0.1% Tween80 on a rotating wheel for two hours, after which, beads with bound material were washed once in 1 mL PBS/0.1% Tween80 in 1.5 mL Eppendorf tubes and then twice in 1 mL PBS. Affinity beads with bound proteins were re-dissolved in 0.2 M TEAB.

### 4.3. Protein Sample Processing for Proteome Analysis

Beads with affinity-purified proteins were on-beads where they were reduced (5 mM dithiotrethiol (DTT), 30 min at 50 °C), alkylated (15 mM iodoacetamide IAA at room temperature in the darkness) and trypsinated (2 µg trypsin, incubated overnight at 37 °C). Protein samples from the immunodepletion experiments and from the ProteoMiner protein equalization experiments were reduced, alkylated, and trypsinated (5 mM DTT, 30 min at 50 °C/15 mM IAA for 30 min at room temperature/2 µg trypsin, incubated overnight at 37 °C).

### 4.4. Nano-LC-MS/MS Analysis

NanoLC-MS/MS analysis was conducted on an Orbitrap Exploris 480 mass spectrometer (Thermo Fisher Scientific, Bremen, Germany) equipped with a nanoHPLC interface (Dionex UltiMate 3000 nano HPLC). The samples (1 µg in 5 µL) were loaded onto a custom made fused capillary pre-column (2 cm length, 360 µm outer OD, 75 µm ID packed with ReproSil Pur C18 3 µm resin (Dr Maish, GmbH)) with a flow of 5µL/min for 8 min. Trapped peptides were separated on a custom made fused capillary column (25 cm length, 360 µm OD,100 µm ID, packed with ReporSil Pur C13 1.9 µm resin) using a linear gradient from 93% solution A (0.1% formic acid) to 29% B (80% acetonitrile in 0.1% formic acid) over 76 min followed by a 3 min gradient to 50% B, 3 min at 90% B, and 7 min at 95% A at a flow rate of 250 nL per minute. Mass spectra were acquired in positive ion mode applying an automatic data-dependent switch between an Orbitrap survey MS scan in the mass range of 350 to 1200 m/z followed by high energy collisional dissociation fragmentation (HCD) and Orbitrap detection of the most intense ions observed in the MS scan in a 2 sec duty cycle. The target value in the Orbitrap for MS scan was 1,000,000 ions at a resolution of 60,000 at m/z 200. For MS2 analysis the automatic gain control (AGC) target mode was set to “standard” with a maximum injection time of 50 milliseconds and a resolution of 15,000. Fragmentation in the ion routing multipole was performed at 30% HCD collision energy. The ion selection threshold was set to 1000 counts. Selected sequenced ions were dynamically excluded for 30 s.

### 4.5. Data Processing

All data files were analyzed in Proteome Discoverer 2.4.0.305 (Thermo Fisher Scientific, Bremen, Germany). The MSPepSearch and Sequest HT processing nodes, both integrated with Proteome Discoverer, were used to search the data with the following cirteria-protein database: UniProt/SwissProt database (downloaded 30/09/2019, containing 42369 sequences) and restricted to humans. The NIST Human Orbitrap spectral library available through Proteome Discoverer were used with MSPepSearch. Fixed search parameters included trypsin, one missed cleavage allowed and carbamidomethylation at cysteine, while methionine oxidation, N-terminal carbamylation and N-terminal acetylation were set as dynamic. Precursor mass tolerance was set to 15 ppm for MSPepSearch and 8 ppm for Sequest HT. Fragment mass tolerance was set to 15 ppm for MSPepSearch and 0.05 Da for Sequest HT. False discovery rate (FDR) was calculated using a decoy database search and only high confidence peptide identifications (FDR < 1%) were included. Label-free quantification was calculated by the Minora feature detector with default settings. Only unique peptides were used for quantification. Correlation plots were created using RStudio (1.2.5001) and R (v.4.0.0) with the pairs.panels() function included in the ‘psych’ package, available through the comprehensive R archive network (CRAN), and correlation coefficients were calculated using the cor() function included in the “stats” package. Venn diagrams were constructed using a publically available software tool (http://bioinformatics.psb.ugent.be/webtools/Venn/).

## 5. Conclusions

In conclusion, we tested four novel chemi-proteomic affinity probes for their ability to enrich for medium- and low-abundant plasma proteins and tested them against three commonly used protein depletion/equalization methods that are traditionally used for plasma biomarker studies. Although complementary to commonly used strategies, our results demonstrated that small-molecule affinity-based methods are excellent alternatives for the analysis of medium-to-low abundant proteins in plasma. This approach offers several advantages over immune depletion methods including scalability to a large number of samples, and targeted detection of very low abundant proteins, and we envisage that this approach will find widespread use in future plasma proteomics experiments.

## Figures and Tables

**Table 1:** The efficiency of the removal of the most abundant 14 plasma proteins by the MARS 14 immunodepletion and ProteoMiner and the four affinity-based approached from human plasma as determined by label free quantification as described in the methods section. Figures shown represent a decrease in signal for the specific protein (in %) after enrichment/depletion, relative to signals obtained from the analysis of un-depleted plasma. Calculation of “% Removed” is based on the mean the signal of six replicate experiments as percentages of the mean of the signal obtained from the analysis of six replicate analysis of un-depleted plasma (see Supplementary Table S1). The concentrations of the measured proteins in plasma is based on values extracted from the plasma proteome database (http://www.plasmaproteomedatabase.org). Abbreviations: agarose-immobilized benzamidine (ABA); O-Phospho-L-Tyrosine (pTYR); 8-Amino-hexyl-cAMP (cAMP) and 8-Amino-hexyl-ATP (ATP); Multi Affinity Removal System (MARS) 14.

Accession Number	Protein Name	[Plasma] µg/mL	% Removed					
			MARS14	Proteominer	ABA	ATP	cAMP	pTYR
P02768	Serum Albumin	42,000	99.8	99.5	98.1	99.8	99.9	99.4
P02671	Fibrinogen alpha chain	3800	72.8	3.7	85.2	99.3	99.5	98.5
P01009	Alpha-1-antitrypsin	3000	99.8	90.5	93.5	98.7	99.5	96.4
P02787	Serotransferrin	2300	99.7	99.1	99.5	99.9	>99.9 ^#^	99.8
P01024	Complement C3	1730	87.3	79.5	96.5	97.3	99.8	98.9
P01023	Alpha-2-macroglobulin	1609	92.7	96.6	98.4	99.2	99.1	99.9
P02647	Apolipoprotein A-I	1400	99.6	<1	95.2	92.9	96.8	89.3
P00738	Haptoglobin	1100	99.1	98.9	97.9	99.5	99.8	98.6
P0DOX5	Immunoglobulin gamma-1 heavy chain	1001	n.d. *	95.1	83.4	95.1	98.1	90.1
P01876	Immunoglobulin heavy constant alpha 1	1000	>99.9 ^#^	92.9	63.4	79.0	86.2	51.5
P02652	Apolipoprotein A-II	780	92.7	51.9	91.3	92.9	91.6	61.4
P02766	Transthyretin	770	<1	<1	94.4	n.d. *	99.3	97.5
P02763	Alpha-1-acid glycoprotein 1	610	99.7	99.9	99.9	n.d. *	n.d. *	n.d. *
P01871	Immunoglobulin heavy constant mu	320	96.1	1.7	<1	<1	<1	<1

n.d. *: Protein not detected in present sample; <1: Signal equals or exceeds signal for un-depleted plasma; >99.9 ^#^: More than 99.95% of this protein is removed.

**Table 2:** Reproducibility of the MARS14 depletion, the Proteominer protein equalization, and the ATP-, cAMP-, pTYR-, and ABA affinity-based enrichment methods. The reproducibility of six replicates of the methods were assessed based precursor ion area detection of identified proteins from individual LC-MS/MS runs and evaluated by linear correlations analysis. The concentrations of the measured proteins in plasma were based on values extracted from the plasma proteome database (http://www.plasmaproteomedatabase.org). The 50 proteins that were annotated with the highest concentrations and the 50 proteins with the lowest concentrations, according to the plasma proteome database, were included in the correlation analysis.

**Correlation Coefficients—50 Most Abundant Plasma Proteins**								
		**Un-Depleted**	**MARS14**	**PM**	**ABA**	**ATP**	**cAMP**	**pTYR**
**Concentration Range [µg/mL]**		42,000–62.36	42,000–47	42,000–57	42,000–47	42,000–41	42,000–15	42,000–27
**Mean**		1.00	0.97	0.93	1.00	0.97	0.97	0.94
**SD**		7.36 × 10^−5^	1.83 × 10^−2^	5.51 × 10^−2^	1.01 × 10^−3^	2.25 × 10^−2^	1.96 × 10^−2^	4.83 × 10^−2^
**Variance**		5.42 × 10^−9^	3.35 × 10^−4^	3.04 × 10^−3^	1.03 × 10^−6^	5.06 × 10^−4^	3.83 × 10^−4^	2.33 × 10^−3^
**Correlation Coefficients—50 Least Abundant Plasma Proteins**								
		**Un-Depleted**	**MARS14**	**PM**	**ABA**	**ATP**	**cAMP**	**pTYR**
**Concentration Range [µg/mL]**		1.1–0.0014	0.025–6.3 × 10 ^−6^	0.02–0.00082	0.0348–0.00082	0.051–0.0011	0.13–0.00082	0.063–0.00082
**Mean**		0.94	0.98	0.83	0.85	0.86	0.99	0.83
**SD**		7.35 × 10^−2^	1.82 × 10^−2^	1.18 × 10^−1^	6.15 × 10^−2^	1.39 × 10^−1^	7.48 × 10^−3^	1.86 × 10^−1^
**Variance**		5.40 × 10^−3^	3.31 × 10^−4^	1.40 × 10^−2^	3.78 × 10^−3^	1.93 × 10^−2^	5.59 × 10^−5^	3.47 × 10^−2^

## Supplementary Materials

Supplementary materials can be found at https://www.mdpi.com/1422-0067/21/16/5903/s1.

## Abbreviations

ABA	para-amino-benzamidine
pTYR	ortho-phospho-L-tyrosine
cAMP	8-amino-hexyl-cyclic adenosine monophosphate
ATP	adenosine triphosphate
MARS14	multiple affinity removal system human 14
LAP	low-abundant protein
MAP	Medium-abundant protein
HAP	high-abundant protein
TEAB	tri ethyl ammonium bisphosphate
IAA	iodoacetamide
HCD	higher energy collisional dissociation

## References

[1] Khoonsari P.E., Haggmark A., Lonnberg M., Mikus M., Kilander L., Lannfelt L., Bergquist J., Ingelsson M., Nilsson P., Kultima K. (2016). Analysis of the Cerebrospinal Fluid Proteome in Alzheimer’s Disease. PLoS ONE.

[2] Anderson N.L., Anderson N.G. (2002). The human plasma proteome: History, character, and diagnostic prospects. Mol. Cell Proteom..

[3] Zhu P., Bowden P., Zhang D., Marshall J.G. (2011). Mass spectrometry of peptides and proteins from human blood. Mass Spectrom. Rev..

[4] Tian R., Jiang X., Li X., Jiang X., Feng S., Xu S., Han G., Ye M., Zou H. (2006). Biological fingerprinting analysis of the interactome of a kinase inhibitor in human plasma by a chemiproteomic approach. J. Chromatogr. A.

[5] Ray S., Reddy P.J., Jain R., Gollapalli K., Moiyadi A., Srivastava S. (2011). Proteomic technologies for the identification of disease biomarkers in serum: Advances and challenges ahead. Proteomics.

[6] Blume J.E., Manning W.C., Troiano G., Hornburg D., Figa M., Hesterberg L., Platt T.L., Zhao X., Cuaresma R.A., Everley P.A. (2020). Rapid, deep and precise profiling of the plasma proteome with multi-nanoparticle protein corona. Nat. Commun..

[7] Zeng Z., Hincapie M., Pitteri S.J., Hanash S., Schalkwijk J., Hogan J.M., Wang H., Hancock W.S. (2011). A proteomics platform combining depletion, multi-lectin affinity chromatography (M-LAC), and isoelectric focusing to study the breast cancer proteome. Anal. Chem..

[8] Sennels L., Salek M., Lomas L., Boschetti E., Righetti P.G., Rappsilber J. (2007). Proteomic analysis of human blood serum using peptide library beads. J. Proteome Res..

[9] Cao Z., Tang H.Y., Wang H., Liu Q., Speicher D.W. (2012). Systematic comparison of fractionation methods for in-depth analysis of plasma proteomes. J. Proteome Res..

[10] Liu X., Valentine S.J., Plasencia M.D., Trimpin S., Naylor S., Clemmer D.E. (2007). Mapping the human plasma proteome by SCX-LC-IMS-MS. J. Am. Soc. Mass Spectrom..

[11] Pernemalm M., Lehtio J. (2013). A novel prefractionation method combining protein and peptide isoelectric focusing in immobilized pH gradient strips. J. Proteome Res..

[12] Zhao Y., Chang C., Qin P., Cao Q., Tian F., Jiang J., Li X., Yu W., Zhu Y., He F. (2016). Mining the human plasma proteome with three-dimensional strategies by high-resolution Quadrupole Orbitrap Mass Spectrometry. Anal. Chim. Acta.

[13] Nanjappa V., Thomas J.K., Marimuthu A., Muthusamy B., Radhakrishnan A., Sharma R., Ahmad K.A., Balakrishnan L., Sahasrabuddhe N.A., Kumar S. (2014). Plasma Proteome Database as a resource for proteomics research: 2014 update. Nucleic Acids Res..

[14] Fratantoni S.A., Piersma S.R., Jimenez C.R. (2010). Comparison of the performance of two affinity depletion spin filters for quantitative proteomics of CSF: Evaluation of sensitivity and reproducibility of CSF analysis using GeLC-MS/MS and spectral counting. Proteom. Clin. Appl..

[15] Qian W.J., Kaleta D.T., Petritis B.O., Jiang H., Liu T., Zhang X., Mottaz H.M., Varnum S.M., Camp D.G., Huang L. (2008). Enhanced detection of low abundance human plasma proteins using a tandem IgY12-SuperMix immunoaffinity separation strategy. Mol. Cell. Proteom..

[16] Ruprecht B., Zecha J., Heinzlmeir S., Medard G., Lemeer S., Kuster B. (2015). Evaluation of Kinase Activity Profiling Using Chemical Proteomics. ACS Chem. Biol..

[17] Bantscheff M., Eberhard D., Abraham Y., Bastuck S., Boesche M., Hobson S., Mathieson T., Perrin J., Raida M., Rau C. (2007). Quantitative chemical proteomics reveals mechanisms of action of clinical ABL kinase inhibitors. Nat. Biotechnol..

[18] Lemeer S., Zorgiebel C., Ruprecht B., Kohl K., Kuster B. (2013). Comparing immobilized kinase inhibitors and covalent ATP probes for proteomic profiling of kinase expression and drug selectivity. J. Proteome Res..

[19] Adachi J., Kishida M., Watanabe S., Hashimoto Y., Fukamizu K., Tomonaga T. (2014). Proteome-wide discovery of unknown ATP-binding proteins and kinase inhibitor target proteins using an ATP probe. J. Proteome Res..

[20] Beavo J.A., Brunton L.L. (2002). Cyclic nucleotide research—Still expanding after half a century. Nat. Rev. Mol. Cell Biol..

[21] Taylor S.S., Kim C., Cheng C.Y., Brown S.H., Wu J., Kannan N. (2008). Signaling through cAMP and cAMP-dependent protein kinase: Diverse strategies for drug design. Biochim. Biophys. Acta.

[22] Scholten A., Poh M.K., van Veen T.A., van B.B., Vos M.A., Heck A.J. (2006). Analysis of the cGMP/cAMP interactome using a chemical proteomics approach in mammalian heart tissue validates sphingosine kinase type 1-interacting protein as a genuine and highly abundant AKAP. J. Proteome Res..

[23] Kaupp U.B., Seifert R. (2002). Cyclic nucleotide-gated ion channels. Physiol. Rev..

[24] Cheng X., Ji Z., Tsalkova T., Mei F. (2008). Epac and PKA: A tale of two intracellular cAMP receptors. Acta Biochim. Biophys. Sin. (Shanghai).

[25] Hofener M., Heinzlmeir S., Kuster B., Sewald N. (2014). Probing SH2-domains using Inhibitor Affinity Purification (IAP). Proteome Sci..

[26] Jankovska E., Svitek M., Holada K., Petrak J. (2019). Affinity depletion versus relative protein enrichment: A side-by-side comparison of two major strategies for increasing human cerebrospinal fluid proteome coverage. Clin. Proteom..

[27] Kim B., Araujo R., Howard M., Magni R., Liotta L.A., Luchini A. (2018). Affinity enrichment for mass spectrometry: Improving the yield of low abundance biomarkers. Expert Rev. Proteom..

[28] Beck H.C., Jensen L.O., Gils C., Ilondo A.M.M., Frydland M., Hassager C., Moller-Helgestad O.K., Moller J.E., Rasmussen L.M. (2018). Proteomic Discovery and Validation of the Confounding Effect of Heparin Administration on the Analysis of Candidate Cardiovascular Biomarkers. Clin. Chem..

