# Supplementary material for: Affinity Capture Enrichment versus Affinity Depletion: A Comparison of Strategies for Increasing Coverage of Low-Abundant Human Plasma Proteins
Source: Int J Mol Sci. 2020 Aug 17;21(16):5903. doi: 10.3390/ijms21165903 (PMC7460666; doi:10.3390/ijms21165903)
Supplement: Supplementary file 1 [file ijms-21-05903-s001.zip › Supplementary Figure 1.pptx]

## Slide 1
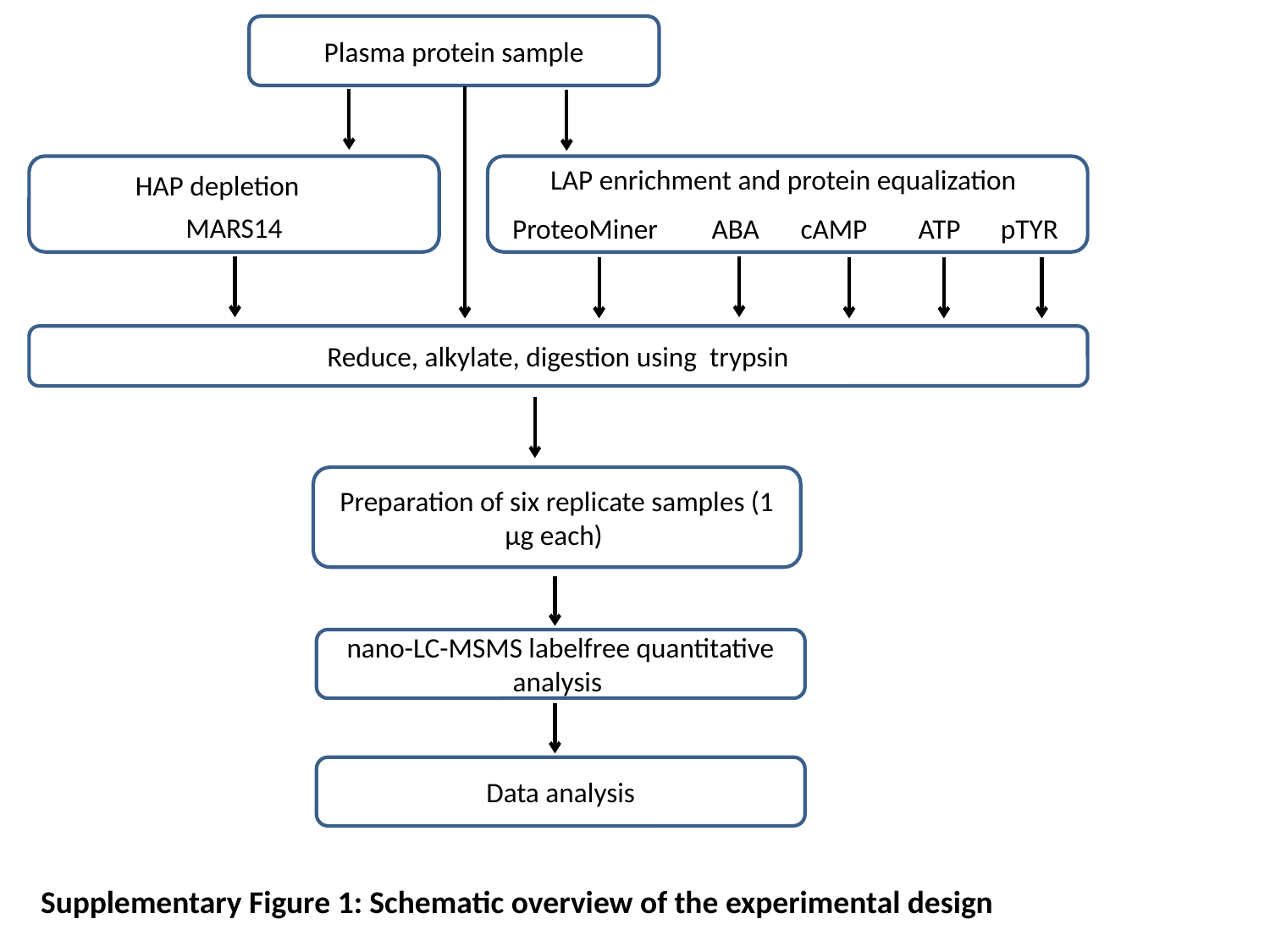

Plasma protein sample
LAP enrichment and protein equalization
HAP depletion
MARS14
ProteoMiner
ABA
cAMP
ATP
pTYR
Reduce, alkylate, digestion using trypsin
Preparation of six replicate samples (1 µg each)
nano-LC-MSMS labelfree quantitative analysis
Data analysis
Supplementary Figure 1: Schematic overview of the experimental design
