# Supplementary figures and images for: Affinity Capture Enrichment versus Affinity Depletion: A Comparison of Strategies for Increasing Coverage of Low-Abundant Human Plasma Proteins
Source: Int J Mol Sci. 2020 Aug 17;21(16):5903. doi: 10.3390/ijms21165903 (PMC7460666; doi:10.3390/ijms21165903)

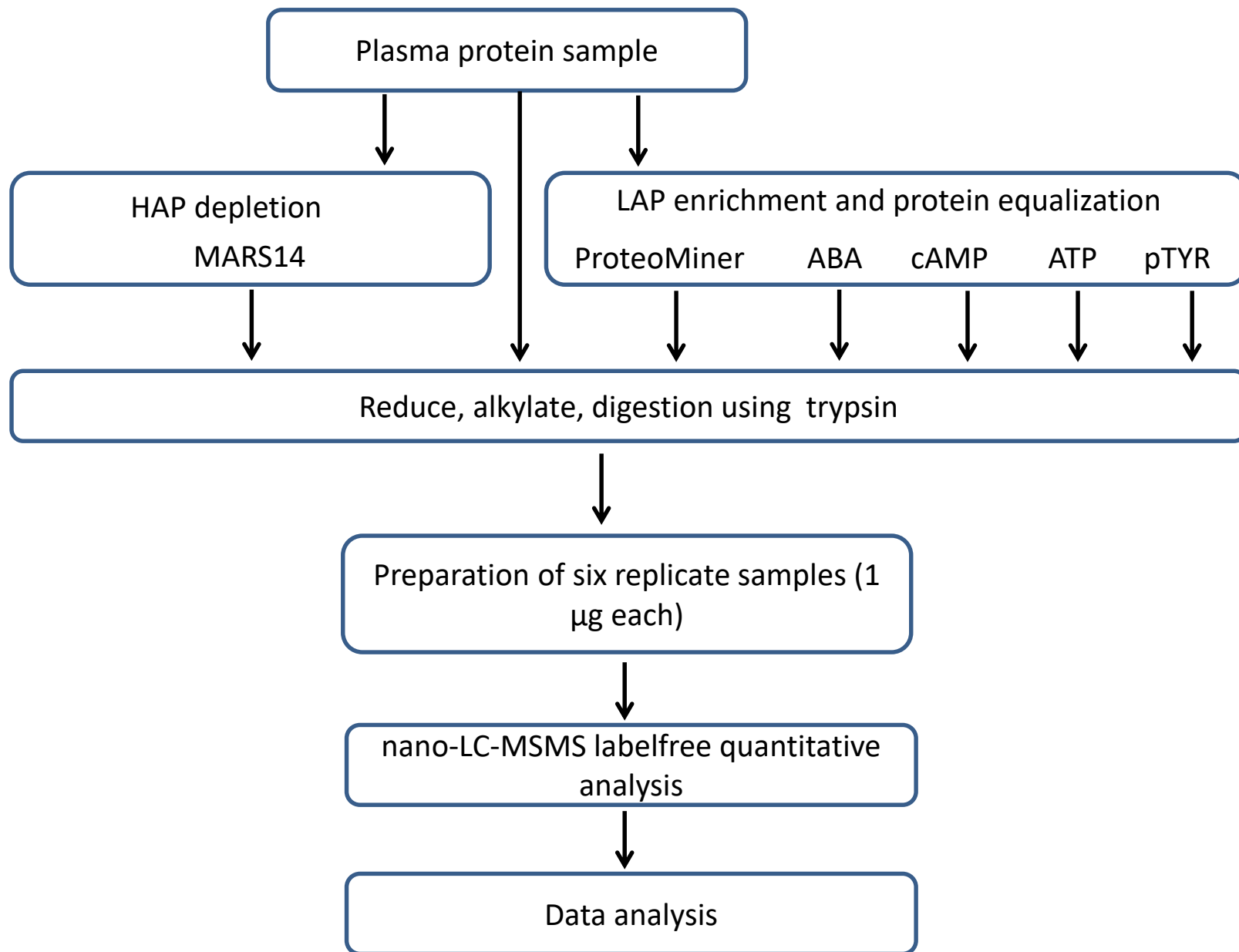

**Supplementary Figure S1: Schematic overview of the experimental design**

Supplement: Supplementary file 1 [file ijms-21-05903-s001.zip › ijms-901550-SUPPL/Supplementary Figure S1.pdf]
